# Insulin Resistance across the Spectrum of Nonalcoholic Fatty Liver Disease

**DOI:** 10.3390/metabo11030155

**Published:** 2021-03-08

**Authors:** Angelo Armandi, Chiara Rosso, Gian Paolo Caviglia, Elisabetta Bugianesi

**Affiliations:** Department of Medical Sciences, University of Turin, 10126 Turin, Italy; angelo.armandi@unito.it (A.A.); chiara.rosso@unito.it (C.R.); gianpaolo.caviglia@unito.it (G.P.C.)

**Keywords:** insulin resistance, NAFLD, cirrhosis, hepatogenous diabetes

## Abstract

Insulin resistance (IR) is defined as a lower-than-expected response to insulin action from target tissues, leading to the development of type 2 diabetes through the impairment of both glucose and lipid metabolism. IR is a common condition in subjects with nonalcoholic fatty liver disease (NAFLD) and is considered one of the main factors involved in the pathogenesis of nonalcoholic steatohepatitis (NASH) and in the progression of liver disease. The liver, the adipose tissue and the skeletal muscle are major contributors for the development and worsening of IR. In this review, we discuss the sites and mechanisms of insulin action and the IR-related impairment along the spectrum of NAFLD, from simple steatosis to progressive NASH and cirrhosis.

## 1. Introduction

Insulin resistance (IR) is defined as a lower-than-expected response to insulin action from target tissues, resulting in the impairment of both glucose and lipid metabolism at different levels and predisposing to the development of type 2 diabetes mellitus (T2DM) [1]. IR is a metabolic abnormality often observed in subjects with nonalcoholic fatty liver disease (NAFLD), and it has been considered one of the major determinants in the pathogenesis of nonalcoholic steatohepatitis (NASH) as well as in the progression of liver disease. The main sites involved in IR are the skeletal muscle, the liver and the adipose tissue; the active crosstalk between these organs is likely to be a major contributor to the development of NAFLD and NASH.

In this review, we discuss the sites and mechanisms of insulin action and IR-related impairment along the spectrum of NAFLD, from simple steatosis to progressive NASH and cirrhosis. 

## 2. Metabolic Effects of Insulin

Insulin is synthesized in pancreatic β-cells of the Langerhans islets as a single chain precursor, pre-proinsulin; subsequently, the removal of a signal peptide in the endoplasmic reticulum generates proinsulin. Proinsulin consists of three domains: an amino-terminal B chain, a carboxy-terminal A chain and a connecting peptide (CP) in the middle. Within the endoplasmic reticulum, proinsulin is exposed to the action of specific endopeptidases that cleave the CP, generating the mature form of insulin. In the postprandial state, when the beta cells are stimulated, insulin is released by exocytosis into the bloodstream, where it maintains blood glucose homeostasis by promoting glucose uptake in several tissues, favoring anabolic metabolism.

The biological action of insulin takes place through the interaction between insulin and its specific receptor. The insulin receptor is a glycosylated tetramer consisting of two extracellular subunits (alpha) and two transmembrane subunits (beta) with tyrosine kinase activity. Insulin binding promotes the autophosphorylation of the receptor and subsequent tyrosine phosphorylation of insulin receptor substrate (IRS) proteins (IRS-1 and IRS-2), which initiate a cascade of events and promote anabolic effects in several organs and tissues [1].

### Sites and Mechanisms of Insulin Action and Insulin Resistance

**Figure 1:**
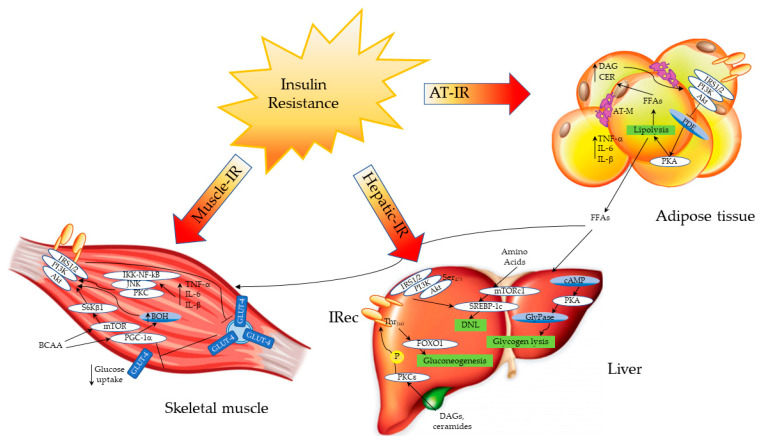
Sites and mechanisms of insulin resistance. Inflamed adipose tissue releases several proinflammatory cytokines such as TNF-α, IL6 and IL-1β, which inhibit the insulin receptor, impairing insulin signaling. In the insulin-resistant condition, the inadequate suppression of lipolysis (due to the impaired inhibition of PKA) promotes the efflux of free fatty acids (FFAs) from the adipose tissue, which reach the liver and the muscle, where they contribute to ectopic fat accumulation. Abbreviations. Adipose tissue insulin resistance (AT-IR); adipose tissue macrophages (AT-M); protein kinase B (Akt); branched-chain amino acids (BCAA); ceramides (CER); DAGs (diacylglycerols); cyclic adenosine monophosphate (cAMP); de novo lipogenesis (DNL); FFAs (free fatty acids); factor forkhead box 01 (FOXO1); glycogen phosphorylase (GlyPase); glucose transporter type 4 (GLUT4); interleukin 1-beta (IL-1β); interleukin 6 (IL-6); insulin receptor (IRec); IRS1/2 (insulin receptor substrate 1/2); protein kinase A (PKA); mammalian target of rapamycin (mTOR); protein kinase C isoform ε (PKCε); phosphodiesterase (PDE); peroxisome proliferator-activated receptor gamma co-activator 1α (PGC-1α); phosphatidylinositol 3 kinase (PI3K); ribosomal protein S6 kinase β1 (S6Kβ1); sterol regulatory element-binding protein-1c (SREBP-1c); tumor necrosis factor alpha (TNF-α).

Several factors, such as hyperinsulinemia and hyperglycemia, together with increased free fatty acid (FFA) concentrations and proinflammatory cytokine levels, may alter insulin signaling in different tissues. These metabolic alterations, which lead to the development and worsening of IR, are common in obese subjects as well as in patients with NAFLD and predispose to the development of T2DM. Despite the primary site of IR being questionable, a growing body of evidence indicates that the periphery plays an important role in the onset of IR, in addition to hepatic steatosis, which exacerbates it [2]. In the skeletal muscle of healthy subjects, insulin activates intracellular signaling through the phosphatidylinositol 3-kinase (PI3K)/protein kinase B (PKB)/mammalian target of rapamycin (mTOR) pathway, thus promoting the expression and translocation of glucose transporter type 4 (GLUT4) from the cytoplasm to the cell membrane. After a glucose load, the absorbed glucose is phosphorylated to glucose-6-phosphate (G6P) by the enzyme hexokinase, and, in this form, it can be used in either the glycolytic or tricarboxylic acid (TCA) pathway, in order to produce energy in the form of adenosine triphosphate (ATP); otherwise, it can be stored as glycogen through glycogen synthesis. In healthy subjects, ~75% of the disposed muscle glucose is converted into glycogen, while 20–25% enters the glycolytic pathway [3]. Moreover, in the skeletal muscle, insulin promotes amino acid (AA) uptake, increases the rate of protein synthesis, and decreases the rate of protein degradation. In the insulin-resistant condition, insulin-mediated glucose uptake in the postprandial state is impaired [3]. In T2DM, the reduction of glycogen synthase activity is an early defect, leading to deranged glycogen synthesis, followed by reduced glucose oxidation [4]. Diabetic patients also show an impaired phosphorylation of IRS-1, leading to a reduced translocation of GLUT4 and a decreased glucose uptake by the muscle [5,6]. IR is associated with a low-grade chronic inflammatory state, which is observed in obese, in diabetic and in NAFLD subjects [7]. Inflammation in myocytes may promote muscle IR through the expression of proinflammatory cytokines such as tumor necrosis factor alpha (TNF-α) and interleukin 1 beta (IL-1β), which in turn activate the protein kinase C (PKC), the c-Jun N-terminal kinase (JNK) and the nuclear factor kappa-light-chain-enhancer of activated B cells (IKK/NF-kB) pathways, thus disrupting insulin signaling [7] (Figure 1).

In the liver, endogenous glucose production is not inhibited due to IR, and glycogen lysis is active, increasing blood glucose levels. The overload of toxic lipids such as DAGs and ceramides activates the PKCε pathway, disrupting insulin signaling and promoting gluconeogenesis through the regulation of FOXO1. Moreover, de novo lipogenesis (DNL) is increased by the regulation of the mTOR and SREBP-1c pathways; branched-chain amino acids (BCAA) can directly activate mTOR, enhancing DNL. In the skeletal muscle, IR impairs the release of GLUT4 from intracellular vesicles to the cell membrane, decreasing glucose uptake. BCAA can activate the mTOR and PGC-1α pathways, affecting the insulin signaling cascade. Similarly, proinflammatory cytokines activate an inflammatory cascade through the PKC/JNK/IKK-NF-kB pathway, disrupting insulin signaling.

Another important cause of IR is the increased amount of FFAs caused by the impaired lipolysis in the adipose tissue. In studies performed both in animal models and in humans, lipid infusion increases diacylglycerol (DAG) levels and PKC signaling, leading to a defective activation of the IRS-1/Akt pathway in the skeletal muscle [8]. An increased lipid content in the myocytes has been observed in type 2 diabetic patients as well as in their offspring. Diacylglycerols, ceramides and long-chain acyl-CoA are directly involved in the development of IR by the inhibition of the Akt pathway, leading to a defective glucose uptake [9,10,11,12]. Recently, Luukkonen P.K. et al. demonstrated that a diet enriched in saturated FFAs increases ceramide levels in overweight nondiabetic subjects, leading to IR by disrupting insulin signaling [13].

Insulin also exerts its activity through branched-chain amino acids (BCAA) (especially leucine), leading to opposite responses: on one hand, leucine can potentiate insulin action through the phosphorylation of IRS and Akt/mTOR [14,15]; on the other hand, high leucine levels can activate mTOR through the ribosomal protein S6 kinase β1 (S6Kβ1), leading to an impairment of insulin signaling, thus promoting IR [16,17]. BCAA can also impair mitochondrial function through the downregulation of peroxisome proliferator-activated receptor gamma co-activator 1α (PGC-1α)-responsive genes, involved in mitochondrial oxidative phosphorylation [18]. Specifically, PGC-1α enhances valine catabolism, leading to the production of the intermediate 3-hydroxyisobuterate (3-BOH) that, in turn, acts as a paracrine factor, reducing insulin sensitivity by inhibiting Akt phosphorylation in C2C12 myotubes [18]. Of note, acylcarnitine, a product of the incomplete oxidation of both BCAA and free fatty acids (FFAs), can induce oxidative stress, thus interfering with insulin signaling [19,20].

In the liver, glucose can be 1. oxidized for energy in the glycolytic pathway, 2. metabolized to CO_2_ and H_2_O through the tricarboxylic acid (TCA) pathway or 3. stored as glycogen via glycogen synthesis. Most of the glucose (about 88%) enters the third metabolic pathway; it is phosphorylated to G6P and contributes to the hepatic glycogen depots [21]. The amount of glycogen in the liver is higher compared to that in the skeletal muscle [21]. During fasting, glucose homeostasis is maintained by hepatic glucose production (HGP), which has two main components: glycogen lysis and gluconeogenesis. The lysis of glycogen is regulated by the enzyme glycogen phosphorylase through the phosphorylation and activation of protein kinase A (PKA) and by the enzyme phosphoglucomutase, which regulates the release of glucose-1-phosphate from the glycogen molecule and its conversion to G6P. After a glucose load, the activation of PKB in the insulin receptor cascade leads to the inhibition of gluconeogenic enzymes via the phosphorylation of FoxO1 and to the inactivation of the enzyme glycogen synthase kinase 3β (GSK3β), which regulates glycogen synthesis [22,23]. When glycogen depots gradually decrease, gluconeogenesis becomes the most important source of glucose [24]. Gluconeogenesis is the synthesis of glucose from noncarbohydrate carbon substrates such as lactate, pyruvate or alanine. It is activated by the induction of the enzymes pyruvate carboxylase (PK) and phosphoenolpyruvate carboxykinase (PEPCK); the latter is inhibited by insulin through Akt/transcription factor forkhead box 01 (FoxO1) phosphorylation. Nuclear magnetic resonance (NMR) 13C spectroscopy combined with the infusion of labelled glucose tracers revealed that after 22, 46 and 64 h of fasting, gluconeogenesis significantly increases from 64% to 82% to 96% and is a major contributor to the HGP in healthy subjects [24].

The excess of glucose is converted into FFAs through the de novo lipogenesis (DNL) pathway, which is regulated by the transcriptional factors sterol regulatory element-binding protein-1c (SREBP-1c) and carbohydrate response element binding protein (ChREBP) by the activation of the transcription of several genes involved in lipogenesis such as acetyl-CoA carboxylase, fatty acid synthetase, acetyl-CoA synthetase and ATP-citrate lyase [25,26]. Diabetic patients are characterized by a defect in glycogen synthesis, which contributes to the increased risk of hypoglycemia during night, and by a 20-fold higher gluconeogenesis when compared to healthy subjects [27]. Several molecular mechanisms play a role in the development of IR in the liver. The impairment of FOXO1 regulation contributes to the increase in gluconeogenesis in patients with T2DM through the increased synthesis of gluconeogenic enzymes [28]. The upregulation of FOXO1 in obese mice leads to IR; conversely, mice knocked out for FOXO-1 show an improvement in insulin sensitivity and normalization of glucose tolerance [29].

Recently, it has been reported that PGC-1α is able to affect IRS-1 and IRS-2 expression, impacting normal glucose homeostasis [30]. Mice fed with a high fat diet show an increase in hepatic DAG content, and DAG can activate PKCε, the primary PKC isoform in the liver [31]. Accordingly, the ectopic lipid accumulation both in the liver and in the muscle, due to adipose tissue IR, leads to the activation of the DAG/PKCε axis in the liver, which in turn inhibits insulin signaling [32]. The association between DAG levels and IR in humans is controversial, and further studies are necessary to understand which lipid species could be considered a signature of IR-associated conditions [33,34].

The adipose tissue is the third major site of insulin action and influences both glucose and lipid homeostasis by releasing adipokines, proinflammatory cytokines and FFAs. The most important role of insulin in the adipose tissue is to suppress lipolysis, a process in which triglycerides are hydrolyzed into glycerol and FFAs, in turn used to provide energy during fasting. Briefly, insulin activates the IRS/PI3K/Akt pathway, thus promoting the phosphorylation of phosphodiesterase 3B, which in turn converts cyclic adenosine monophosphate (cAMP) into 5′-AMP. The reduction in cAMP decreases the phosphorylation of PKA and reduces the lipolysis rate [35]. Insulin-stimulated glucose disposal in the adipose tissue is negligible when compared to that in the skeletal muscle, but FFAs that are released into the bloodstream impact glucose homeostasis, exacerbating muscle IR.

The second metabolic pathway regulated by insulin in the adipose tissue is lipogenesis, which leads to the accumulation of triglycerides in the adipocytes [27]. Excess food intake and energy storage leads to hypertrophic and inflamed adipose tissue. Several proinflammatory factors such as TNF-α, IL-6 and IL-1β are overexpressed in enlarged adipocytes compared to smaller ones, linking hypertrophic obesity to IR [36,37]. TNF-α promotes the serine phosphorylation of IRS-1, decreasing its association with PI3K, thus disrupting insulin signaling [38] (Figure 1). In the IR condition, the activation of PKA by cAMP leads to the phosphorylation of hormone-sensitive lipase (HSL) and perilipin; the subsequent translocation of HSL from the cytosol to the lipid droplet surface enhances lipolysis. The result of the impaired suppression of lipolysis is an increased release of FFAs and glycerol into the bloodstream and ectopic fat accumulation [38]. Among ectopic lipids, toxic species such as DAG and ceramides can disrupt insulin signaling by inhibiting insulin receptor and Akt activation [39]. Adipose tissue IR has also been linked to mitochondrial dysfunction and mitophagy; decreased mitochondrial biogenesis and reduced mitochondrial oxidative protein content lead to a reduced oxidative capacity [40,41].

## 3. Insulin Resistance in Nonalcoholic Fatty Liver Disease

### 3.1. Relation between Hepatic Steatosis and Insulin Resistance

The association between NAFLD and IR has been widely investigated. The prevalence of IR is high in NAFLD and even higher in subjects with NASH compared to those with simple steatosis [42]; to date, IR is considered the main pathogenetic mechanism involved in the onset of NAFLD and its progression to NASH [43,44,45]. In the IR state, fat accumulation in the liver is caused by the impaired uptake, synthesis, export and oxidation of FFAs. In NAFLD subjects, the amount of hepatic steatosis correlates with increased plasma levels of FFAs due to the impaired suppression of lipolysis from the adipose tissue; subcutaneous adipose tissue represents a major source of intrahepatic fat (~62–82% of intrahepatic triglycerides). This mechanism is independent of obesity and diabetes, as it has also been demonstrated in nonobese, nondiabetic NAFLD patients; in the latter group, IR affects HGP, glucose disposal (glycogen synthesis and glucose oxidation), lipolysis and lipid oxidation. Although visceral fat is not the main supplier of circulating FFAs, it represents the main source of inflammatory cytokines reaching the liver, as confirmed by the correlation between IL-6 and C-reactive protein levels in the portal vein [46,47].

In the insulin-resistant condition, the liver loses its ability to suppress HGP in response to insulin and enhances DNL through the activation of the Notch signaling pathway [48]. This explains, on one hand, the increase in DNL that, in NAFLD patients, is 5-fold higher when compared to that in healthy subjects (26 vs. 5%, respectively) and, on the other hand, the predisposition to diabetes in subjects with NAFLD [48].

### 3.2. Insulin Resistance in the Progression from Simple Steatosis to Nonalcoholic Steatohepatitis and Fibrosis

The development of NASH has been linked to a variety of factors, including nutrient intake, endocrine derangements (insulin, leptin, adiponectin and ghrelin), alterations in gut microbiota (endotoxemia) and epigenetic factors, possibly acting on a genetic predisposition [49]. Unfortunately, the molecular mechanisms leading to NASH and fibrosis development have not been fully elucidated yet. Hepatic triglyceride depositions into lipid droplets are considered a sort of inert storage; notwithstanding this, excessive lipid overload may enhance lipid oxidation and reactive oxygen species (ROS) release, making the liver susceptible to the action of proinflammatory factors. In obese and in diabetic patients, ROS levels correlate with the C-reactive protein concentration as well as fibrinogen levels, suggesting a subclinical proinflammatory state [49,50]. In NAFLD, the saturated FFA palmitate seems to play an important role in the progression of liver damage; it is synthesized in the DNL pathway and is able to trigger fibrogenesis in the liver through the activation of hepatic macrophages [51].

Several factors are able to mediate liver damage in patients with NAFLD; some of these are synthesized by the liver, while others are released by the adipose tissue and exert their effects in a paracrine way [52]. The liver is the main source of selenoprotein P (SeP), a selenium carrier protein with antioxidant properties. SeP is regulated by hyperglycemia and is able to induce IR, disrupting glucose homeostasis, thus favoring the development of T2DM [52]. Recently, we found that circulating SeP increases according to the degree of hepatic steatosis and to the stage of fibrosis in nondiabetic patients with NAFLD, suggesting its potential role in the onset of NASH and progression to fibrosis [53].

Leptin is a peptide hormone that is released by adipocytes and plays a role in the regulation of food intake and bodyweight. In the setting of NAFLD, leptin may be expressed by activated hepatic stellate cells (HSCs) and by Kupffer cells (KCs), contributing to hepatic fibrogenesis, thus enhancing HSC signal transduction [49,54]. Specifically, leptin stimulates the transcriptional activation of both the α1(I) and α2(I) fibrils, which are major components of dense extracellular matrix (ECM). Furthermore, leptin promotes the synthesis of the matrix metalloproteinase-2 (MMP-2), tissue inhibitor matrix metalloproteinase 1 (TIMP-1), TIMP-2, and alpha-smooth muscle actin (α-SMA) transcripts, all involved in the pathogenesis of liver fibrosis [55,56]. Finally, leptin protects HSCs against apoptosis [55]. Although higher leptin levels were found in patients with NAFLD compared to healthy controls, its role in the pathogenesis of NASH has not been fully elucidated.

Insulin-like growth factor 1 (IGF-1) is a hormone very similar to insulin in its molecular structure. IGF-1 is expressed primarily by the liver under the control of growth hormone, and it circulates linked to IGF-binding protein 3 (IGFBP-3). IGF-1 is involved in hepatocyte differentiation, proliferation and apoptosis [57]. A recent meta-analysis showed that IGF-1 levels are reduced in NAFLD patients compared to healthy controls, suggesting a potential role as a therapeutic target [58]. Moreover, Hagstrom et al. found low IGF-1 levels in patients with severe fibrosis (F ≥ 3) compared to those with absent/mild fibrosis [59]. Even though the molecular mechanisms linking IGF-1 and the progression of liver damage in the setting of NAFLD have not been elucidated yet, recent data describe a novel role of IGF-1 in regulating stress-induced hepatocyte premature senescence in liver fibrosis. Specifically, IGF-1 is able to attenuate the oxidative stress-induced premature senescence of hepatocytes in mice through the inhibition of the interaction between nuclear p53 and progerin, a truncated version of the lamin A protein, improving hepatic steatosis and fibrogenesis [60].

The liver is the main target for adiponectin, the most abundant adipocytokine synthesized by the adipose tissue [61]. Low adiponectin levels are associated with steatosis, inflammation and fibrosis in the liver [61]. Specifically, circulating adiponectin decreases in obese subjects as well as in fibrotic patients with NAFLD. Adiponectin exerts an antifibrotic action by reducing HSC activation and proliferation; in addition, it favors matrix degradation, reducing the molecular ratio of MMP-1 to TIMP-1, antagonizing leptin-mediated signaling in hepatic fibrogenesis [62]. This potent profibrogenic effect of leptin may contribute to the endothelial alteration of hepatic sinusoids, whose fenestrations are progressively replaced by an organized basement membrane. This process, known as the “capillarization” of hepatic sinusoids, is one of the major parenchymal alterations that drive the liver to an architecture causing portal hypertension [63,64].

In the past few years, genome-wide association studies have led to the identification of several genes related to NAFLD, NASH, and their complications including hepatocellular carcinoma (HCC). In 2008, Romeo et al. [65] described a single-nucleotide polymorphism (SNP) in the patatin-like phospholipase domain-containing 3 (*PNPLA3*) gene, which encodes the triglyceride lipase adiponutrin and strongly affects fat accumulation in the liver through mechanisms independent of IR. The *PNPLA3* rs738409 (G) risk allele, found in ~40% of the European population, can also increase, threefold, the risk of progression to NASH and, most importantly, twelve-fold, the risk of developing HCC [66].

In subsequent years, other SNPs have been associated with increased hepatic fat accumulation and, thus, the progression of liver disease. The most important genetic variants are the rs58542926 C > T located in the transmembrane 6 superfamily member 2 (*TM6SF2*) gene and the rs641738 C > T located in the membrane-bound O-acyl-transferase domain-containing 7 (*MBOAT7*) gene, which favor hepatic fat accumulation in intracellular lipid droplets via different mechanisms, increasing the susceptibility to inflammation, NASH and fibrosis [67].

## 4. Insulin Resistance in Advanced Liver Disease

### 4.1. Insulin Resistance and Cirrhosis

Liver cirrhosis represents the final stage of the natural history of any chronic liver disease. The parenchymal structure becomes progressively subverted by regenerative nodules and fibrosis septa. Its course is indolent, until portal hypertension develops from persistent splanchnic hemodynamic changes and the disruption of intrahepatic sinusoids. Portal hypertension paves the way to systemic damage and the onset of clinical manifestations of liver disease. In addition, the presence of cirrhosis itself constitutes the proinflammatory ground where hepatocellular carcinoma (HCC) can develop, along with etiology-driven additional damage.

**Figure 2:**
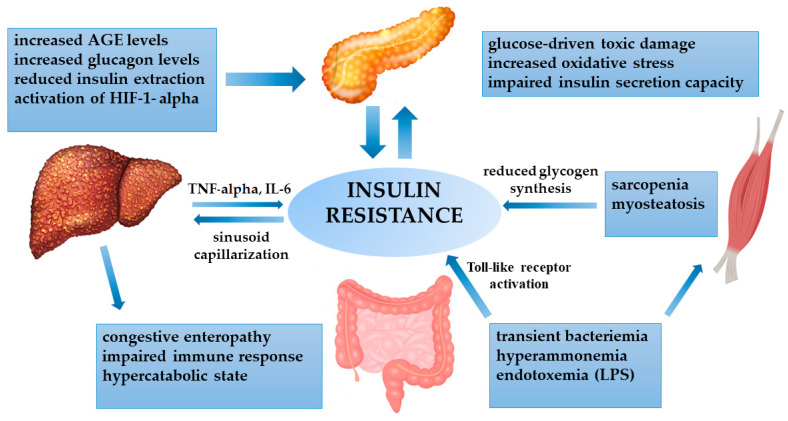
Crosstalk between the cirrhotic liver and peripheral tissues in determining insulin resistance. Abbreviations. Advanced glycation end product (AGE); hypoxia-inducible factor 1-alpha (HIF-1α); interleukin-6 (IL-6); lipopolysaccharide (LPS); tumor necrosis factor-alpha (TNF-α).

In patients with NAFLD-related cirrhosis, IR represents the primum movens of the chronic liver disease. On the other hand, it is known that all cirrhotic patients are prone to being insulin resistant, irrespective of the etiology, because cirrhosis itself may lead to alterations in glucose metabolism [68,69,70]. Nevertheless, IR as assessed by the homeostasis model of assessment of insulin resistance (HOMA-IR) index may differ according to the cause of cirrhosis, being higher in NAFLD- and HCV-related chronic hepatitis, compared to alcohol- and HBV-related disease [71]. Specific hepatokines can contribute to IR, such as selenoprotein-P, which has a direct influence on insulin action in skeletal muscle in NAFLD subjects [72]. Furthermore, cytokines secreted by the liver, as a consequence of the persistent necroinflammatory activity, such as TNF-α and IL-6, can also induce IR [73]. In cirrhotic patients, multiple distortions are involved in the onset of IR (Figure 2).

In cirrhosis, impaired hepatocyte functionality and portal hypertension cause reduced insulin extraction, with subsequent hyperinsulinemia. In addition, glucagon is not properly metabolized, with persistent hyperglycemia. The cirrhosis-related chronic inflammatory state worsens insulin resistance by the secretion of cytokines (TNF-α and IL-6) and by enhancing a persistent hypoxic state, with the activation of HIF-1α. In return, the increased insulinemia contributes to sinusoid capillarization, favoring portal hypertension. The cirrhosis-induced hypercatabolic condition and congestive enteropathy cause hyperammonemia and impaired gut permeability, with subsequent endotoxemia and the activation of Toll-like receptors. These factors directly impact insulin sensitivity, but also skeletal muscle activity, leading to sarcopenia and fat infiltration (myosteatosis), with reduced glycogen synthesis. Advanced glycation end products (AGEs) are not properly metabolized by the liver, with increased toxic damage to the pancreas. Finally, all the derangements impact the pancreas, where oxidative stress and glucose toxicity cause progressive damage to beta cells, whose impairment is crucial for developing overt diabetes.

The augmented blood levels of insulin in end-stage liver disease are an effect of liver function impairment. Moreover, portal hypertension is thought to be another crucial driver of hyperinsulinemia. Due to the parenchymal alterations in advanced cirrhosis, the splanchnic blood flow cannot be properly conveyed throughout the liver. Hence, the blood is pumped in secondary small vessels that drive the flow from the portal vein to the systemic circulation. These vessels constitute the so-called “portosystemic shunts” that are responsible for conveying metabolites inside the systemic circulation, bypassing hepatocyte extraction. Likewise, high levels of insulin, coming from the splanchnic circulation, are not properly metabolized inside the liver and directly cause systemic hyperinsulinemia.

The two counterparts work in parallel and with a synergic effect. In mouse models undergoing partial hepatectomy, insulin levels dramatically increase [74], even in the absence of significant portal hypertension; when portosystemic shunts are suppressed in advanced liver disease, insulin clearance is ameliorated, suggesting a role of portal hypertension in determining IR [75]. Similarly, contra-insulin hormones, such as glucagon, are not adequately metabolized by damaged hepatocytes and persistently stimulate glucose production.

One of the most typical features of cirrhotic patients is malnutrition, due to both decreased nutrient intake and diminished protein synthesis in a persistent catabolic state. The reduction of liver glycogen leads to an accelerated fasting condition and a parallel increase in gluconeogenesis, mainly driven by amino acids from muscle proteins. All these factors inevitably lead to skeletal muscle wasting and sarcopenia, a well-known hallmark of cirrhosis associated with poor prognosis [76].

Increased levels of ammonia are a common finding in advanced liver disease, depending on both impaired hepatocyte metabolism and portosystemic shunts. Hyperammonemia directly affects skeletal muscle, stimulating the synthesis of myostatin. Myostatin seems to be crucial in cirrhosis-induced peripheral IR, as it depletes protein mass and induces fat accumulation inside the muscle. This phenomenon, known as “myosteatosis”, can downregulate muscle insulin receptor, leading to impaired glucose transport and glycogen synthesis [77,78].

The transient bacteremia in cirrhosis is due to the congestion of the small and large intestine in portal hypertension with altered permeability. A impaired immune response facilitates pathogenic strain proliferation [79] and the translocation of gut-derived endotoxins (mainly lipopolysaccharide), leading to the activation of Toll-like receptors, involved in the pathogenesis of IR [80].

The deterioration of beta-cell function is mainly driven by chronic hyperglycemia, which causes toxic damage to the pancreatic islets of Langerhans [81,82,83]. This process is enhanced by the accumulation of advanced glycation end products (AGEs), by inducing oxidative stress. The liver is involved in the clearance of AGEs; in cirrhosis, these products may not be properly metabolized, thus boosting oxidative stress in beta cells [84]. One further worsening factor is constituted by the systemic low-grade hypoxia induced by cirrhosis and related to the severity of the disease [85]. Chronic liver tissue injury and the persistence of alterations in normal intrahepatic vascular perfusion are responsible for the hypoxic parenchymal microenvironment. Moreover, the ineffective liver clearance of vasodilating agents (e.g., nitric oxide), coming from the splanchnic circulation, may affect pulmonary regulatory function, resulting in diffusion–perfusion defects and persistent capillary dilation, contributing to systemic hypoxemia.

The subsequent activation of hypoxia-inducible factors (mainly HIF-1α) can trigger an inflammatory response in beta cells, as well as directly altering glucose metabolism [86], contributing to the development of overt diabetes.

### 4.2. Hepatogenous Diabetes

About 80% of cirrhotic patients become glucose intolerant, and nearly 20% of them develop frank diabetes [87]. In 1906, diabetes arising in cirrhosis was renamed “hepatogenous diabetes” to distinguish this entity from type 2 diabetes [88]. Hepatogenous diabetes shares neither the same risk factors of type 2 diabetes (family history, obesity and older age) nor the same complications (mainly micro- and macrovascular damage) [89]. The onset of hepatogenous diabetes is relatively more rapid compared to that of type 2 diabetes, as up to 20% of cirrhotic patients develop hyperglycemia within 5 years [90,91]. This form of diabetes often presents with normal glucose levels (due to impaired glucose metabolism) and normal glycated hemoglobin (due to the reduced lifespan of erythrocytes) [92,93]. As a result, the presence of diabetes can only be suspected based on high glycated hemoglobin levels in a small proportion of patients. Interestingly, fructosamine measurement, which reflects the glycemic status over 2–4 weeks, seems to be more accurate for the evaluation of glycemic control in this population [94].

In hepatogenous diabetes, the specific pathophysiological pathways include impaired insulin sensitivity, which represents an early event, and subsequently beta-cell dysfunction, essential for the transition to frank diabetes [88].

The interplay between peripheral tissues and the liver is crucial in determining the phenotype. One seminal Australian study conducted in 1980 found that the liver production of endogenous glucose was markedly reduced in cirrhotic patients, even in those without diabetes, as a consequence of glucose intolerance driven by peripheral (muscle) IR, while the liver was hypersensitive to insulin action [95]. One subsequent study from Petrides et al. demonstrated a reduction of glycogen synthesis in the muscle [96]. As cirrhosis progresses, there is no further worsening of IR: in one study conducted in 2016 on 300 pretransplant patients, the proportions of individuals with diabetes, with respect to those without diabetes or with prediabetes, did not differ among the different grades of cirrhosis severity [97].

Hepatogenous diabetes does not affect short-term survival but seems to be associated with higher mortality in long-term periods [98,99,100], mainly driven by portal hypertension rather than the micro-/macrovascular complications of diabetes. Indeed, few patients develop retinopathy, as well as cardiovascular events [89].

Different longitudinal studies conducted on cirrhotic patients have assessed the role of diabetes in reducing transplantation-free survival (Table 1), with discordant results.

In one French study including 348 decompensated patients, diabetes was independently correlated with worse survival in patients with better liver functionality, as expressed by a Model for End-stage Liver Disease (MELD) score lower than 10 [103]. However, another large study conducted for a median time of 17 years challenged the independent impact of diabetes on survival [101].

Nonetheless, diabetes can impact the onset and severity of specific liver-related complications. Ascites seems to occur more frequently when diabetes is present, irrespectively of residual liver functionality [104]. This may be related to the microvascular alterations caused by diabetes that occur in the kidney and in the liver, facilitating the onset of portal hypertension [75,105]. Similarly, diabetes has been associated with hepatic encephalopathy, independently of MELD score [106]. Autonomic neuropathy and impaired intestinal motility may accelerate the onset of small intestinal bacterial overgrowth and bacterial translocation, a major causal factor of encephalopathy. Interestingly, the administration of acarbose in patients with cirrhosis and diabetes significantly reduced blood ammonia levels, improving psychometric tests for minimal encephalopathy [107]. Diabetes acts in synergy with cirrhosis in conferring a higher risk of bacterial infections, as emerged from studies conducted on patients undergoing liver transplantation [108].

The impact of diabetes on HCC is complex. In one large meta-analysis of 28 prospective studies including cirrhotic patients, diabetes was associated with an increased incidence of HCC and HCC-related mortality. Pre-existent diabetes, rather than hepatogenous diabetes, appeared to have a well-defined impact on the onset of HCC [109]. This is crucial when considering HCC in NAFLD-related cirrhosis, where metabolic derangements are the main drivers of both liver damage and carcinogenesis, even in noncirrhotic NAFLD patients [110].

Liver transplantation may improve or reverse diabetes, but this positive effect is counterbalanced by the susceptibility to developing diabetes due to immunosuppressive therapy and changes in nutritional habits [111,112,113]. Some studies have reported a successful regression of pre-existent diabetes in up to two thirds of cases [114], whereas other authors have found a substantial lack of improvement at one-year follow-ups, as shown by a markedly low insulin response during the oral glucose tolerance test (OGTT) [113]. Abnormalities in glucose tolerance seem to persist after liver transplantation even in patients with apparently normal glucose profiles [115]. Grancini et al. proved the central role of beta-cell functionality in explaining such discrepancies. In fact, rescued beta-cell functionality after transplantation allowed an increase in insulin bioavailability, playing a central role in favoring diabetes regression [116]. On the other hand, beta cells’ irreversible secretory defects are mainly responsible for the inefficacy of liver transplantation regarding the improvement of diabetes [116].

## 5. Conclusions

Insulin resistance is a common feature in NAFLD subjects, and it is considered one of the most important “hits” driving the progression from simple steatosis to NASH along with lifestyle, genetic predisposition and gut microbiota changes. In the liver, lipid overload enhances oxidative stress, leading to mitochondrial dysfunction, which in turn exacerbates inflammation and activates inflammatory pathways.

Insulin resistance is also a common finding in cirrhotic patients, irrespective of etiology. Glucose metabolism disturbances are not easily detected, due to multiple systemic perturbations. Careful monitoring is required, particularly of beta-cell residual functionality, which is crucial for the transition to overt diabetes and potential reversal after liver transplantation. Frank diabetes should be actively managed, as it may impact long-term survival and the severity of liver-related events.

## Figures and Tables

**Table 1:** Longitudinal studies conducted on cirrhotic patients evaluating the impact of diabetes on liver-related events and mortality. * Decompensated cirrhosis; ** not significant according to multivariate analysis.

Study	Year of Publication	Number of Patients	Follow-Up (Months)	Outcome
Bianchi et al. [98]	1984	382 *	37	death (HR = 2.30, *p* = 0.019)
Moreau et al. [99]	2004	75 *	18	death (HR = 2.20, *p* = 0.03)
Sangiovanni et al. [101]	2006	214	204	not significant
Berman et al. [102]	2011	447 *	3	not significant
Quintana et al. [100]	2011	110	30	death (OR = 3.30, *p* = 0.007) **
Elkrief et al. [103]	2014	348 *	60	ascites (OR = 1.70, *p* = 0.05)
				bacterial infections (OR = 3.02, *p* = 0.001)
				HE (OR = 6.55, *p* < 0.001)
				HCC (OR = 1.93, *p* = 0.016)
				death (HR = 1.33, *p* = 0.027)

**Abbreviations**. Hepatocellular carcinoma (HCC); hepatic encephalopathy (HE); hazard ratio (HR); odds ratio (OR).
